# The Resurgent Structure of Quantum Knot Invariants

**DOI:** 10.1007/s00220-021-04076-0

**Published:** 2021-06-08

**Authors:** Stavros Garoufalidis, Jie Gu, Marcos Mariño

**Affiliations:** 1 International Center for Mathematics, Department of Mathematics, Southern University of Science and Technology, Shenzhen, China; 2 Département de Physique Théorique, Université de Genève, 1211 Geneva 4, Switzerland; 3 Section de Mathématiques et Département de Physique Théorique, Université de Genève, 1211 Geneva 4, Switzerland

## Abstract

The asymptotic expansion of quantum knot invariants in complex Chern–Simons theory gives rise to factorially divergent formal power series. We conjecture that these series are resurgent functions whose Stokes automorphism is given by a pair of matrices of *q*-series with integer coefficients, which are determined explicitly by the fundamental solutions of a pair of linear *q*-difference equations. We further conjecture that for a hyperbolic knot, a distinguished entry of those matrices equals to the Dimofte–Gaiotto–Gukov 3D-index, and thus is given by a counting of BPS states. We illustrate our conjectures explicitly by matching theoretically and numerically computed integers for the cases of the $4_1$ and the $5_2$ knots.

## Introduction

### Asymptotic expansions in perturbative quantum field theory

Perturbative expansions in quantum field theories are often mathematically defined but typically lead to factorially divergent formal power series. Important examples are the perturbative expansions of the partition function of 3-dimensional manifolds (with, or without boundary) in complex Chern–Simons theory with arbitrary gauge group. For instance, in [GLMn08] it was shown that the LMO invariant of a 3-manifold (which is the perturbative expansion of the Witten–Reshetikhin–Turaev invariant at the trivial flat collection and arbitrary gauge group), is a Gevrey-1 formal power series, that is a formal power series whose *n*th coefficient is bounded by $n! C^n$ for some positive constant *C*. Said differently, these perturbative expansions have Borel transforms which are germs of holomorphic functions at the origin.

In [Gar08] it was conjectured that the perturbative expansions in complex Chern–Simons theory are resurgent functions, and more precisely that they have analytic continuation as multivalued functions in the complex plane minus a discrete and computable set of points, placed in finitely many vertical lines in the complex plane. These vertical lines are formed by an infinite towers of singularities, with a $2 \pi \mathrm{i}$ periodicity. The position of these singularities is dictated by the values of the complex Chern–Simons function (a $\mathbb {C}/2 \pi \mathrm{i} \mathbb {Z}$-valued function) on the set of flat connections.

### Resurgence in complex Chern–Simons theory

In what follows, we will identify the partition function of complex Chern–Simons theory with the state integral of Andersen-Kashaev in [AK14], following the ideas of Hikami, Dimofte et al [Hik07, DGLZ09]. Although this identification has not been derived from first principles, it turns out to have a number of startling consequences. We will focus on manifolds of the form $M= {\mathbb {S}}^3 \backslash {\mathcal {K}}$, where ${\mathcal {K}}$ is a hyperbolic knot.

Our goal is to give an explicit description of the resurgent structure of the formal power series of perturbative Chern–Simons theory in terms of a fundamental solution of a pair of linear *q*-difference equation and a matrix of integers. We will describe the general story first, and illustrate it with concrete examples later.

We will denote by ${\mathcal {P}}$ the set of critical points of the complex Chern–Simons action and by $\sigma$ a typical critical point. Given our identification of Chern–Simons theory with state integrals, it turns out that the set ${\mathcal {P}}$ coincides with the set of critical points of the integrand of the state integral, an effectively computable set of algebraic numbers. The critical values of the complex Chern–Simons function are labeled by $\sigma$ and an integer $\mu$ (often called “multicovering”):

1 $$\begin{aligned} \text {CS}(\sigma ;\mu ) = {V(\sigma ) \over 2\pi } - 2\pi {\mathrm{i}} \mu , \qquad \mu \in \mathbb {Z}. \end{aligned}$$

We conjecture that the corresponding transseries $\Phi _{\sigma , \mu }$ satisfy the translation invariance property

2 $$\begin{aligned} \Phi _{\sigma ,\mu }(\tau ) = {\tilde{q}}^\mu \, \Phi _{\sigma }(\tau ), \qquad {\tilde{q}}= \mathrm{e}^{-2\pi \mathrm{i}/\tau }, \end{aligned}$$

where $\Phi _{\sigma }(\tau )$ is the conventional asymptotic expansion of the state integral around the saddle point $\sigma$. It has the form

3 $$\begin{aligned} \Phi _{\sigma }(\tau )= \exp \left( { V(\sigma ) \over 2\pi \tau } \right) \varphi _\sigma (\tau ), \qquad \varphi _\sigma (\tau ) \in \mathbb {C}[[\tau ]]. \end{aligned}$$

As a consequence, all the Stokes automorphisms acting on $\Phi _{\sigma ,\mu }$ are packaged in two Stokes automorphism matrices $\mathsf {S}^{+}(q)$, $\mathsf {S}^-(q^{-1})$, which are matrices of *q*- and $q^{-1}$-series respectively, and each of which encodes Stokes automorphism across a half-plane. Their detailed definition is given in Sect. 5.1.

An important feature of state integrals is that they depend on additional parameters and this leads to a system of a pair of linear *q*-difference equations, one in the upper half-plane and another in the lower half-plane [GK17]. In our examples, these linear *q*-difference equations have explicit sets of fundamental solutions. We conjecture that

#### Conjecture 1

- $\mathsf {S}^{\pm }(q)$ are bilinear functions of two fundamental solutions of the pair of linear *q*-difference equations.
- $\mathsf {S}^{\pm }(q)$ satisfy the inversion relation
  4 $$\begin{aligned} \mathsf {S}^+(q)^T \mathsf {S}^-(q) = \mathbf {1} \,. \end{aligned}$$
- $\mathsf {S}^{\pm }(q)$ are uniquely determined by $\mathsf {S}^+(0)$, $\mathsf {S}^-(0)$ and a pair of fundamental solutions to the pair of linear *q*-difference equations.

The above matrices $\mathsf {S}^{\pm }$ uniquely determine the collection of transseries $\Phi _{\sigma ,\mu }(\tau )$ for all $(\sigma ,\mu )$ via an abstract Riemann-Hilbert correspondence first pointed out in [Vor83] and developed recently in [GMN10, IMnS19, KS20]. Note that this transcendental correspondence converts the difficult problem of computing coefficients of $\Phi _{\sigma ,\mu }(\tau )$ (typically, one can not compute more than a couple of hundred coefficients) into the much easier problem of computing fundamental solutions of linear *q*-difference equations, up to a matrix of unknown integers.

Given a hyperbolic knot ${\mathcal {K}}$ there is a distinguished critical point $\sigma _1$ (the geometric representation, corresponding to the complete hyperbolic structure), and in that case we conjecture a precise relation between the entry $\mathsf {S}^+_{\sigma _1,\sigma _1}(q)$ of the matrix $\mathsf {S}^+(q)$ and the (rotated) 3D-index of Dimofte–Gaiotto–Gukov [DGG14, DGG13].

#### Conjecture 2

We have:

5 $$\begin{aligned} \mathsf {S}^+_{\sigma _1\sigma _1}(q) = \,\mathrm {Ind}_{\mathcal {K}}^\mathrm {rot}(q)\,. \end{aligned}$$

We recall that the 3D-index ${\mathcal {I}}_{\mathcal {K}}(m,e)(q)$ associated to a knot ${\mathcal {K}}$ is labeled by two integers (*m*, *e*). It counts BPS states in a three-dimensional, ${\mathcal {N}}=2$ supersymmetric theory $T_M$ which can be associated to the manifold $M={\mathbb {S}}^3\backslash {\mathcal {K}}$ [DGG14]. The rotated index is then given by

6 $$\begin{aligned} \mathrm {Ind}_{\mathcal {K}}^\mathrm {rot}(q)=\sum _{e \in {\mathbb {Z}}} {\mathcal {I}}_{\mathcal {K}}(0,e)(q). \end{aligned}$$

The relation in (5) between the resurgent structure of complex Chern–Simons theory and a counting of BPS states in the corresponding supersymmetric theory was anticipated in [Mn19, GGMn20b].

We emphasize that although the state integrals and their perturbation theory are well-defined, the above picture is largely conjectural. However, it fits well with the work of Kontsevich–Soibelman [KS11], as well as with a lecture of Kontsevich on June 30, 2020 [Kon20], and it paves the way for a deeper understanding of the topological/physical meaning of the integers appearing in $\mathsf {S}^{\pm }$.

We should point out that the above theory in fact has little to do with knots and 3-manifolds and complex Chern–Simons theory, and little to do with the Bloch group, but appears to be part of a larger combinatorial structure. This is apparent in the data needed to define the formal power series of [DG13, DG18] as well as the data needed to define *q*-hypergeometric Nahm sums (and thus their asymptotic expansion at roots of unity [GZa]) and the data needed to define state integrals [GK17]. This combinatorial structure is sometimes called a $K_2$-Lagrangian, or an extended symplectic group.

We will illustrate the above conjectures concretely for the invariants of the two simplest hyperbolic knots, the $4_1$ and the $5_2$ knots. Some aspects of the resurgent structure of complex Chern–Simons theory for the $4_1$ knot were studied in [GMnP, GH18], but they focused on the “classical" transseries $\Phi _{\sigma }(\tau )$ (i.e. they didn’t address the resurgent structure of the tower of singularities). The resurgent problem in the case of compact $\mathrm {SU}(2)$ Chern–Simons theory was addressed in [CG11, GMnP], where complete towers of Stokes constants were explicitly computed for some Seifert three-manifolds.

This paper is, in a sense, a sequel to [GZc] and [GZb] which the reader can consult for further information, motivation, historical presentation, as well as for the connection with the asymptotics of the Kashaev invariant and with the quantum modularity conjecture.

Note that our notation $\varphi _\sigma (\tau )$ from Equation (3) corresponds to the notation $\Phi ^{(\sigma )}_0(2\pi \mathrm{i}x)$ of [GZc]. In particular, the coefficient of $\tau ^n$ in $\varphi _\sigma (\tau )$ is (up to multiplication by an eighth root of unity and the square root of an nonzero element of $F_\sigma$) in $(2\pi \mathrm{i})^n F_\sigma$, where $F_\sigma$ is the trace field of $\sigma$.

## The Equation ${(1-x)(1-x^{-1})}$ = 1 and the $4_1$ Knot

The state integral of the $4_1$ knot is given by Eq. (39) below with $(A,B)=(1,2)$ and $\mu =\lambda =0$. The critical points of the integrand are solutions of the algebraic equation

7 $$\begin{aligned} (1-x)(1-x^{-1})=1. \end{aligned}$$

The latter has two solutions $\xi _1= \mathrm{e}^{2 \pi \mathrm{i}/6}$ and $\xi _2= \mathrm{e}^{-2 \pi \mathrm{i}/6}$ which lie in the number field $\mathbb {Q}(\sqrt{-3})$, the trace field of the $4_1$ knot. The corresponding series $\Phi _{\sigma _j}(\tau )$ satisfy the relation $\Phi _{\sigma _2}(\tau ) = \mathrm{i} \Phi _{\sigma _1}(-\tau )$ and the first few terms of $\varphi _{\sigma _1}(\tau /(2\pi \mathrm{i})) \in 3^{-1/4} \mathbb {Q}(\sqrt{-3})[[\tau ]]$ are given by

8 $$\begin{aligned} \varphi _{\sigma _1}\left( \frac{\tau }{2\pi \mathrm{i}}\right) = \frac{1}{\root 4 \of {3}}\, \Bigl (1 \,{+}\, \frac{11\tau }{72\sqrt{-3}} \,{+}\, \frac{697\tau ^2}{2\,(72\sqrt{-3})^2} \,{+}\, \frac{724351\tau ^3}{30\,(72\sqrt{-3})^3} \,{+}\,\cdots \Bigr )\,. \end{aligned}$$

The exponent in (3) involves the hyperbolic volume of the $4_1$ knot complement

9 $$\begin{aligned} V(\sigma _1)= V=2 \mathrm{Im}\, \mathrm{Li}_2(\mathrm{e}^{\mathrm{i}\pi /3})= 2.029883\dots \,. \end{aligned}$$

The two series $\Phi _{\sigma _{j}}(\tau )$ for $j=1,2$ form a vector

10 $$\begin{aligned} \Phi (\tau )= \begin{pmatrix} \Phi _{\sigma _1}(\tau )\\ \Phi _{\sigma _2}(\tau ) \end{pmatrix} \end{aligned}$$

that also appears in the refined quantum modularity conjecture [GZc]. $\Phi (\tau )$ is the vector of series whose description in Borel plane we wish to give.

Consider the linear *q*-difference equation

11 $$\begin{aligned} y_{m+1}(q) -(2-q^m) y_m(q) + y_{m-1}(q)=0 \qquad (m \in \mathbb {Z}) \,. \end{aligned}$$

It has a fundamental solution set given by the columns of the following matrix

12 $$\begin{aligned} W_m(q) = \begin{pmatrix} G^0_m(q) &{} G^1_m(q) \\ G^0_{m+1}(q) &{} G^1_{m+1}(q) \end{pmatrix}, \end{aligned}$$

where $G^0_m(q)$ and $G^1_m(q)$ are defined by

13a $$\begin{aligned} G^0_m(q)&=\sum _{n=0}^\infty (-1)^n \frac{q^{n(n+1)/2+m n}}{(q;q)_n^2} \end{aligned}$$

13b $$\begin{aligned} G^1_m(q)&=\sum _{n=0}^\infty (-1)^n \frac{q^{n(n+1)/2+m n}}{(q;q)_n^2} \left( 2m+ E_1(q) + 2 \sum _{j=1}^n \frac{1+q^j}{1-q^j} \right) \,, \end{aligned}$$

and $E_1(q)=1-4\sum _{n=1}^\infty q^n/(1-q^n)$ is the Eisenstein series.

It is easy to see that $G^0_m$ satisfies (11). Indeed, $G^0_m(q)=\sum _{n=0}^\infty a_{n,-m}(q)^{-1}$ where $a_{n,m}(q)$ is given in (34) below. Equations (35a)–(35c), applied to $a_{n,-m}(q)^{-1}$ conclude the result. A similar proof applies for $G^1_m$. Another way to do so is to use the state integral (39) (with $(A,B)=(1,2)$) and show that the latter satisfies the linear *q*-difference Eq. (11) in two ways, one with respect to the variable $\lambda$ and another with respect to the variable $\mu$.

The fundamental solution $W_m(q)$ satisfies

14 $$\begin{aligned} \det (W_m(q))=2 \end{aligned}$$

and the symmetry

15 $$\begin{aligned} W_m(q^{-1}) = W_{-m}(q) \begin{pmatrix} 1 &{} 0 \\ 0 &{} -1 \end{pmatrix} \,, \end{aligned}$$

and the orthogonality

16 $$\begin{aligned} \frac{1}{2} W_m(q) \begin{pmatrix} 0 &{} 1 \\ -1 &{} 0 \end{pmatrix} W_{m}(q)^T = \begin{pmatrix} 0 &{} 1 \\ -1 &{} 0 \end{pmatrix} \,. \end{aligned}$$

for all integers *m*, as well as

17 $$\begin{aligned} \frac{1}{2} W_m(q) \begin{pmatrix} 0 &{} 1 \\ -1 &{} 0 \end{pmatrix} W_{\ell }(q)^T \in \mathrm {SL}(2,\mathbb {Z}[q,1/q]) \end{aligned}$$

for all integers $m, \ell$. The $\mathsf {S}$ matrix is given by

18a $$\begin{aligned} \mathsf {S}^+(q)&={1\over 2} \begin{pmatrix} 0 &{} 1 \\ 1 &{} 1 \end{pmatrix} W_{-1}(q) \begin{pmatrix} 0 &{} 1 \\ 1 &{} 0 \end{pmatrix}W_{-1}(q)^T \begin{pmatrix} 0 &{} -1 \\ 1 &{} 2 \end{pmatrix}, \end{aligned}$$

18b $$\begin{aligned} \mathsf {S}^-(q)&= {1\over 2} \begin{pmatrix} -1 &{} -1 \\ 0 &{} 1 \end{pmatrix} W_{-1}(q) \begin{pmatrix} 0 &{} 1 \\ 1 &{} 0 \end{pmatrix}W_{-1}(q)^T \begin{pmatrix} 1 &{} 0 \\ -2 &{} 1 \end{pmatrix} \,. \end{aligned}$$

The above matrix $\mathsf {S}$ satisfies Eq. (4).

## The Equation $x^{-2}(1-x)^3$ = 1 and the $5_2$ Knot

The state integral of the $5_2$ knot is given by Eq. (39) below with $(A,B)=(2,3)$ and $\mu =\lambda =0$. The critical points of the integrand are solutions of the algebraic equation

19 $$\begin{aligned} x^{-2}(1-x)^3=1 \,. \end{aligned}$$

The above equation (which defines a cubic field of discriminant $-23$, the trace field of the $5_2$ knot) has three solutions $\xi _1=0.78492+1.30714\dots \mathrm{i}$, $\xi _2=0.78492-1.30714\dots \mathrm{i}$ and $\xi _3=0.43016\dots$, corresponding to the geometric representation, its conjugate and the real representation. The corresponding series $\Phi _{\sigma _j}(\tau )$ satisfy $\Phi _{\sigma _2}(\tau ) = -\mathrm{i}{\overline{\Phi }}_{\sigma _1}(-\tau )$ and the first few terms of $\phi _{\sigma _j}(\tau /(2\pi \mathrm{i}))$ are given by

20 $$\begin{aligned}&\varphi _{\sigma _j}\left( \frac{\tau }{2\pi \mathrm{i}}\right) = \left( \frac{-3\xi _j^2+3\xi _j-2}{23}\right) ^{1/4}\nonumber \\&\quad \left( 1+\frac{33\xi _j^2+242\xi _j-245}{2^2\cdot 23^2}\tau +\frac{100250\xi _j^2-12643\xi _j+2732}{2^5\cdot 23^3}\tau ^2\right. \nonumber \\&\quad +\frac{-50198891\xi _j^2+35443870\xi _j-79016748}{2^7\cdot 3\cdot 5\cdot 23^5}\tau ^3 \nonumber \\&\quad \left. +\frac{-3809943572\xi _j^2+1861268771\xi _j+1015686665}{2^{11}\cdot 3\cdot 5 \cdot 23^6}\tau ^4+\dots \right) \,. \end{aligned}$$

The exponent in (3) involves

21 $$\begin{aligned} V(\sigma _1) = 2.821\ldots + 1.379\ldots \mathrm{i},\; V(\sigma _2) = -2.821\ldots + 1.379\ldots \mathrm{i},\; V(\sigma _3) = -2.758\ldots \quad \end{aligned}$$

where $\mathfrak {R}\,V(\sigma _1)$ is the hyperbolic volume of the $5_2$ knot complement, and $\mathfrak {I}\,V(\sigma _1) = \mathfrak {I}\,V(\sigma _2)$ the Chern–Simons action. The three series $\Phi _{\sigma _j}(\tau )$ for $j=1,2,3$ form a vector

22 $$\begin{aligned} \Phi (\tau ) = \left( \begin{array}{c} \Phi _{\sigma _1}(\tau )\\ \Phi _{\sigma _2}(\tau )\\ \Phi _{\sigma _3}(\tau ) \end{array} \right) . \end{aligned}$$

Consider the linear *q*-difference equation

23 $$\begin{aligned} y_m(q)-3y_{m+1}(q)+(3-q^{2+m})y_{m+2}(q) - y_{m+3}(q) = 0. \end{aligned}$$

The above equation has a fundamental solution set given by the columns of the following matrix

24 $$\begin{aligned} W_m(q) = {\left\{ \begin{array}{ll} W_m^+(q),\quad &{}|q|<1,\\ \begin{pmatrix} 0&{}0&{}1\\ 0&{}1&{}0\\ 1&{}0&{}0 \end{pmatrix} W_{-m-2}^-(q^{-1}) \begin{pmatrix} 1&{}0&{}0\\ 0&{}-1&{}0\\ 0&{}0&{}1 \end{pmatrix},\quad&|q|>1. \end{array}\right. } \end{aligned}$$

where the matrices $W_m^\varepsilon (q)$ with $\varepsilon =\pm$ are respectively

25 $$\begin{aligned} W^\varepsilon _m(q) = \begin{pmatrix} H^{\varepsilon }_{0,m}(q) &{} H^{\varepsilon }_{1,m}(q) &{} H^{\varepsilon }_{2,m}(q) \\ H^{\varepsilon }_{0,m+1}(q) &{} H^{\varepsilon }_{1,m+1}(q) &{} H^{\varepsilon }_{2,m+1}(q) \\ H^{\varepsilon }_{0,m+2}(q) &{} H^{\varepsilon }_{1,m+2}(q) &{} H^{\varepsilon }_{2,m+2}(q) \end{pmatrix} \end{aligned}$$

and $H^\varepsilon _{j,m}(q)$ are given in “Appendix A” for $j=0,1,2$ and $m \in \mathbb {Z}$.

The fundamental solutions satisfy

26 $$\begin{aligned} \det (W_m(q))=2, \end{aligned}$$

for all integers *m* as well as

27 $$\begin{aligned} \frac{1}{2} W_m(q) \begin{pmatrix} 0&{}0&{}1 \\ 0&{}2&{}0 \\ 1&{}0&{}0 \end{pmatrix} W_{\ell }(q^{-1})^T \in \mathrm {SL}(3,\mathbb {Z}[q,1/q]) \end{aligned}$$

for all integers $m, \ell$. In particular, we have:

28 $$\begin{aligned} \frac{1}{2} W_{m-1}(q) \begin{pmatrix} 0&{}0&{}1 \\ 0&{}2&{}0 \\ 1&{}0&{}0 \end{pmatrix} W_{-m-1}(q^{-1})^T = \begin{pmatrix} 1&{}0&{}0 \\ 0&{}0&{}1 \\ 0&{}1&{}3-q^{m} \end{pmatrix} \,. \end{aligned}$$

The $\mathsf {S}$ matrix is given by

29a $$\begin{aligned} \mathsf {S}^+(q)&= \frac{1}{2} \begin{pmatrix} 0&{}1&{}0\\ 0&{}1&{}1\\ -1&{}0&{}0 \end{pmatrix} W_{-1}(q^{-1}) \begin{pmatrix} 0&{}0&{}1\\ 0&{}-2&{}0\\ 1&{}0&{}0 \end{pmatrix} W_{-1}(q)^T \begin{pmatrix} 0&{}0&{}-1\\ 1&{}1&{}0\\ 0&{}1&{}0 \end{pmatrix}, \end{aligned}$$

29b $$\begin{aligned} \mathsf {S}^-(q)&= \frac{1}{2} \begin{pmatrix} 0&{}3&{}-1\\ 0&{}-1&{}0\\ 1&{}0&{}0 \end{pmatrix} W_{-1}(q) \begin{pmatrix} 0&{}0&{}1\\ 0&{}-2&{}0\\ 1&{}0&{}0 \end{pmatrix} W_{-1}(q^{-1})^T \begin{pmatrix} 0&{}0&{}1\\ 3&{}-1&{}0\\ -1&{}0&{}0 \end{pmatrix}\,. \end{aligned}$$

The above matrix $\mathsf {S}$ satisfies Eq. (4). A proof is given in “Appendix A.2”.

## Descendants

A key aspect of our study of asymptotic series are linear *q*-difference equations which are satisfied for their descendants. This elementary idea leads to descendants of the Kashaev invariant (studied extensively in [GZc]), of asymptotic series (ibid), of *q*-series as well as of state integrals. In this section we review in detail the story of descendants (or ancestors, as the case may be).

### The Kashaev invariant and its descendants

The series $\Phi _{\sigma _1} (\tau )$ appearing in the saddle-point expansion of the state integral appeared originally in the asymptotic expansion of the Kashaev invariant [Kas95]. In the case of the $4_1$ knot, the Kashaev invariant is given by

30 $$\begin{aligned} {\mathbf {J}}^{(4_1)}(q) = \sum _{n=0}^\infty (q;q)_n (q^{-1};q^{-1})_n \,. \end{aligned}$$

The above expression can be evaluated when *q* is a root of unity. The Volume Conjecture of Kashaev [Kas97] (and its extension to all orders [Guk05]) asserts that ${\mathbf {J}}^{(4_1)}(\mathrm{e}^{2 \pi \mathrm{i}/N})$ has an asymptotic expansion for *N* large of the form

31 $$\begin{aligned} {\mathbf {J}}^{(4_1)}(\mathrm{e}^{2 \pi \mathrm{i}/N}) \sim N^{3/2}\, \Phi _{\sigma _1}\left( \frac{1}{N}\right) . \end{aligned}$$

We now explain a relation discovered in [GZc] between the formula for the Kashaev invariant (30) and the algebraic Eq. (7).

Following [GZc], we define the descendants ${\mathbf {J}}^{(4_1)}_m(q)$ of the Kashaev invariant of the $4_1$ knot by

32 $$\begin{aligned} {\mathbf {J}}^{(4_1)}_m(q) = \sum _{n=0}^\infty (q;q)_n (q^{-1};q^{-1})_n \, q^{m n}, \qquad (m \in \mathbb {Z}) \,. \end{aligned}$$

Then, the sequence ${\mathbf {J}}^{(4_1)}_m(q)$ is a solution to a linear *q*-difference equation

33 $$\begin{aligned} q^{m+1} {\mathbf {J}}_m(q) + (1-2q^m){\mathbf {J}}_m(q) + q^{m-1} {\mathbf {J}}_{m-1}(q) =1, \qquad (m \in \mathbb {Z}) \,. \end{aligned}$$

This can be seen as follows: let

34 $$\begin{aligned} a_{n,m}(q)= (q;q)_n (q^{-1};q^{-1})_n q^{m n} \end{aligned}$$

denote the summand of (32). It follows that

35a $$\begin{aligned} a_{n+1,m}(q)&= (1-q^{-n-1})(1-q^{n+1}) \,a_{n,m}(q) \end{aligned}$$

35b $$\begin{aligned}&= q^m(2-q^{n+1}-q^{-n-1}) \, a_{n,m}(q) \end{aligned}$$

35c $$\begin{aligned}&= q^m(2-q \, a_{n,m+1}(q)-q^{-1} a_{n,m-1}(q)) \,. \end{aligned}$$

Summing over $n \ge 0$ and taking into account the boundary term $a_{0,m}(q)=1$ on the left hand side of the above equation concludes the proof of Eq. (33).

Using the operators *E* and *Q* that act on sequences $(y_m)$ by

36 $$\begin{aligned} (Ey)_m=y_{m+1}, \qquad (Qy)_m=q^m y_m, \qquad EQ=qQE \end{aligned}$$

it follows that we can write (33) in the form

37 $$\begin{aligned} ( Q(1-qE)(1-q^{-1}E^{-1}) - I) {\mathbf {J}}_m(q) = 1 \,. \end{aligned}$$

The homogeneous part of the above operator can be obtained by replacing *x* in the left hand side of Eq. (7) *x* by *qE*, and replacing the right hand side of Eq. (7) by $Q^{-1}$.

### The *q*-series $(G^0_0,G^1_0)$ and their descendants

We now discuss an appearance of the formal power series $\Phi (\tau )$ in the radial asymptotics of some *q*-series, following [GZb].

By *q*-series we mean formal Laurent series in a variable *q* with integer coefficients, i.e., elements of $\mathbb {Z}((q))$. All the *q*-series below will define holomorphic functions in the punctured unit disk with (perhaps) a pole at the origin. We now recall how the radial asymptotics of the *q*-series $(G^0_0,G^1_0)$ is given by $\Phi (\tau )$. The first series $G^0_0(q)$ was found quite by accident to have radial asymptotics expressed in terms of the series $\Phi (\tau )$ [GZb], whereas the second series was found systematically by expressing the state integral invariant of the $4_1$ knot in terms of products of *q*-series and ${\tilde{q}}$-series [GK17].

Below, we will use capital letters for *q*-series and small letters for the corresponding functions on the upper half-plane, e.g., $g^0_m(\tau )=G^0_m(q)$ for $q=\mathrm{e}^{2\pi \mathrm{i}\tau }$. In [GZb] it was observed that we have an asymptotic expansion

38 $$\begin{aligned} \begin{pmatrix} \frac{1}{\sqrt{\tau }} g^0_0(\tau ) \\ \sqrt{\tau } g^1_0(\tau ) \end{pmatrix} \sim \begin{pmatrix} 1 &{} -1 \\ 1 &{} 1 \end{pmatrix} \Phi ( \tau ) \end{aligned}$$

to all orders in $\tau$, as $\tau$ tends to 0 along a ray in the first quadrant of the upper half-plane. The above asymptotic expansion requires some explanation since on a fixed ray in the first quadrant, $\Phi _{\sigma _1}( \tau )$ is exponentially larger than $\Phi _{\sigma _2}(\tau )$. Nonetheless, the asymptotic expansion (38) makes sense theoretically and computationally if we use a refined optimal truncation explained in detail in [GZc] and applied in [GZb]. The numerical computations of [GZb] hinted that the matrix in Eq. (38) is the constant term of a matrix of ${\tilde{q}}$ series.

Given the definition of $G^0_0(q)$ and $G^1_0(q)$ from [GZb] and [GK17], it was relatively straightforward to add the variable $q^{m n}$ and arrive to formulas (13a) and (13b) which define the descendants of the pair $(G^0_0(q),G^1_0(q))$.

### The state integral and its descendants

In this section we recall the definition of state integrals and some of their basic properties. State integrals are multidimensional integrals whose integrand is a product of Faddeev’s quantum dilogarithm function $\Phi _{\mathsf {b}}$ (whose definition we will not need and may be found in [Fad95, FK94]) times an exponential of a quadratic and linear form. Here $\tau =\mathsf {b}^2 \in \mathbb {C}':=\mathbb {C}{\setminus }(-\infty ,0]$, thus even if the integrand contains no free variables, a state integral is always a holomorphic function of $\tau$.

State integrals have two key properties:

- They define holomorphic functions in the complex cut plane $\mathbb {C}'$.
- They can be expressed bilinearly in the upper and in the lower half-plane in terms of products of *q*-series and ${\tilde{q}}$-series.

For a detailed discussion of state integrals and numerous example, see for instance [BDP14] and also [AK14] and [GK17].

In this section we introduce a descendant version of the one dimensional state integrals of [GK17] that satisfies the above properties. In this section we will use the notation from [GK17]. Consider the state integral

39 $$\begin{aligned} {\mathcal {I}}_{A,B,\lambda ,\mu }(\mathsf {b}) = \int _{\mathbb {R}+\mathrm{i}\epsilon } \Phi _{\mathsf {b}}(x)^B \mathrm{e}^{-A\pi \mathrm{i}x^2 + 2\pi (\lambda \mathsf {b}- \mu \mathsf {b}^{-1})x}\mathrm{d}x,\qquad (\lambda ,\mu \in \mathbb {Z}) \end{aligned}$$

where *A* and *B* are integers and $B> A > 0$. Under these assumptions, it follows that the integrand is exponentially decaying at infinity and the integral is absolutely convergent and defines a holomorphic function of $\tau = \mathsf {b}^2 \in \mathbb {C}'$. Below, we will use the notation $\phi (w,\delta _\bullet )$, ${\tilde{\phi }}(w,{\tilde{\delta }}_\bullet )$ and

40 $$\begin{aligned} {\langle F(q,x) \rangle } = F(q,1) \end{aligned}$$

from [GK17].

#### Theorem 3

Fix integers *A* and *B* with $B> A > 0$ and integers $\lambda$ and $\mu$. For all $\tau$ with $\mathfrak {I}(\tau )>0$, we have:

41 $$\begin{aligned} {\mathcal {I}}_{A,B,\lambda ,\mu }(\mathsf {b}) = (-1)^{\lambda -\mu } q^{\frac{\lambda }{2}}{\tilde{q}}^{\frac{\mu }{2}} \left( \frac{{\tilde{q}}}{q}\right) ^{\frac{B-3A}{24}} \mathrm{e}^{\pi \mathrm{i}\frac{B+2(A+1)}{4}} {\langle P_{A,B,\lambda ,\mu } \left( F_{A,B,\lambda }(q,x)\widetilde{F}_{A,B,\mu }({\tilde{q}},{\tilde{x}})\right) \rangle }\nonumber \\ \end{aligned}$$

where the operator $P_{A,B,\lambda ,\mu }$ is given by

42 $$\begin{aligned} P_{A,B,\lambda ,\mu } = \mathrm {Res}_{w=0} \left( \mathrm{e}^{\frac{w^2}{4\pi \mathrm{i}}+w\left( \mathsf {b}(\delta +1/2+\lambda /A) +\mathsf {b}^{-1}({\tilde{\delta }}+1/2-\mu /A)\right) } \right) ^A \left( \frac{\phi (\mathsf {b}w,\delta _\bullet ) {\tilde{\phi }}(\mathsf {b}^{-1}w,{\tilde{\delta }}_\bullet )}{\mathsf {b}(1-\mathrm{e}^{\mathsf {b}^{-1}w})}\right) ^B .\nonumber \\ \end{aligned}$$

In particular, the right hand side of Eq. (41) is a bilinear combination of *q* and ${\tilde{q}}$-series extend to the cut plane $\mathbb {C}'$. A similar formula can be given when $\tau$ is in the lower half-plane, and what is more, the state integral satisfies the symmetry ${\mathcal {I}}_{A,B,\lambda ,\mu }(\mathsf {b}) = {\mathcal {I}}_{A,B,\lambda ,\mu }(\mathsf {b}^{-1})$ which is a consequence of the symmetry $\Phi _{\mathsf {b}^{-1}}(x)=\Phi _{\mathsf {b}}(x)$ of the quantum dilogarithm.

#### Proof

We follow the derivation in [GK17] closely. The idea is to sum up residues at all singularities in the upper half-plane. The factor $\Phi _{\mathsf {b}}(x)$ has poles at

43 $$\begin{aligned} x_{m,n} = \mathrm{i}\mathsf {b}(m+1/2) + \mathrm{i}\mathsf {b}^{-1}(n+1/2),\quad m,n\in \mathbb {N}\,. \end{aligned}$$

We notice that

44 $$\begin{aligned} \mathrm{e}^{2\pi (\lambda \mathsf {b}- \mu \mathsf {b}^{-1})(x+x_{m,n})} = \mathrm{e}^{w(\lambda \mathsf {b}- \mu \mathsf {b}^{-1})} q^{\lambda (m+1/2)}{\tilde{q}}^{\mu (n+1/2)}(-1)^{\lambda -\mu } \end{aligned}$$

where we have made the change of variables $w = 2\pi x$ and used the notation

45 $$\begin{aligned} q = \mathrm{e}^{2\pi \mathrm{i}\mathsf {b}^2},\quad {\tilde{q}}= \mathrm{e}^{-2\pi \mathrm{i}\mathsf {b}^{-2}}. \end{aligned}$$

Now by modifying Eq. (27) of [GK17], we find

46 $$\begin{aligned}&{\mathcal {I}}_{A,B,\lambda ,\mu }(\mathsf {b}) = (-1)^{\lambda -\mu } q^{\frac{\lambda }{2}}{\tilde{q}}^{\frac{\mu }{2}} \left( \frac{{\tilde{q}}}{q}\right) ^{\frac{B-3A}{24}} \mathrm{e}^{\pi \mathrm{i}\frac{B+2(A+1)}{4}} \nonumber \\&\quad \times \sum _{m,n=0}^\infty \left( \mathrm {Res}_{w = 0}F_{A,B,m,n,\lambda ,\mu }(w)\right) \frac{q^{\lambda m}t_m(q)^A}{(q;q)_m^B} \frac{{\tilde{q}}^{\mu n}{\tilde{t}}_n({\tilde{q}})^A}{({\tilde{q}}^{-1};{\tilde{q}}^{-1})_n^B} \,, \end{aligned}$$

where

47 $$\begin{aligned} F_{A,B,m,n,\lambda ,\mu }(w) = \left( e^{\frac{w^2}{4\pi \mathrm{i}}+w\left( \mathsf {b}(m+1/2 +\lambda /A)+\mathsf {b}^{-1}(n+1/2-\mu /A)\right) } \right) ^A \left( \frac{\phi _m(\mathsf {b}w){\tilde{\phi }}_n(\mathsf {b}^{-1}w)}{\mathsf {b}(1-e^{\mathsf {b}^{-1}w})}\right) ^{B}.\nonumber \\ \end{aligned}$$

Using the operator formalism in [GK17], this concludes the proof of Eq. (41). $\square$

The reader may find in [GK17] the expressions of the operators $\phi (w,\delta _\bullet )$, ${\tilde{\phi }}(w,{\tilde{\delta }}_\bullet )$. Note that $e_l({\tilde{q}})$ in the paper are simply $E_l^{(0)}({\tilde{q}})$. In addition,

48 $$\begin{aligned}&F_{A,B,\lambda }(q,x) = \sum _{n=0}^\infty (-1)^{A n} \frac{q^{A\frac{n(n+1)}{2}+n\lambda }}{(q;q)_n^B} x^n, \end{aligned}$$

49 $$\begin{aligned}&\widetilde{F}_{A,B,\mu }({\tilde{q}},{\tilde{x}}) = \sum _{n=0}^\infty (-1)^{(B-A) n} \frac{{\tilde{q}}^{(B-A)\frac{n(n+1)}{2}+n\mu }}{({\tilde{q}};{\tilde{q}})_n^B} {\tilde{x}}^n. \end{aligned}$$

They can be related by

50 $$\begin{aligned} F_{A,B,\lambda }(q^{-1},x) = \widetilde{F}_{A,B,-\lambda }(q,x). \end{aligned}$$

#### Example 4

In this example we illustrate Theorem 3 with the state integral ${\mathcal {I}}_{1,2,\lambda ,\mu }(\mathsf {b})$ associated to the $4_1$ knot [AK14]. As we will see, this reproduces the *q*-series $G^0_m(q)$ and $G^1_m(q)$ of (13a) and (13b).

Using

51 $$\begin{aligned} F_\lambda (q,x) = F_{1,2,\lambda }(q,x) = \widetilde{F}_{1,2,\lambda }(q,x) = \sum _{n=0}^\infty (-1)^n \frac{q^{\frac{n(n+1)}{2}+n\lambda }}{(q;q)_n^2}x^n. \end{aligned}$$

we find that

52 $$\begin{aligned} P_{1,2,\lambda ,\mu } = \frac{\mathsf {b}}{2}(1+2\delta - 4\delta _1+2\lambda ) -\frac{\mathsf {b}^{-1}}{2}(1+2{\tilde{\delta }} - 4{\tilde{\delta }}_1 + 2\mu ). \end{aligned}$$

It then follows that ($\tau = \mathsf {b}^2$)

53 $$\begin{aligned} {\mathcal {I}}_{1,2,\lambda ,\mu }(\mathsf {b}) = (-1)^{\lambda -\mu +1}\mathrm{i}q^{\frac{\lambda }{2}}{\tilde{q}}^{\frac{\mu }{2}} \left( \frac{q}{{\tilde{q}}}\right) ^{\frac{1}{24}} \left( \frac{\tau ^{1/2}}{2}G^1_\lambda (q)G^0_\mu ({\tilde{q}}) - \frac{\tau ^{-1/2}}{2}G^0_\lambda (q)G^1_\mu ({\tilde{q}})\right) ,\nonumber \\ \end{aligned}$$

where we have used that

54 $$\begin{aligned} {\langle F_{\lambda }(q,x) \rangle }=G^0_\lambda (q) , \qquad {\langle (1+2\lambda +2\delta -4\delta _1)F_\lambda (q,x) \rangle }=G^1_\lambda (q). \end{aligned}$$

In “Appendix A”, we give the details for the state integral ${\mathcal {I}}_{2,3,\lambda ,\mu }(\mathsf {b})$ of the $5_2$ knot.

## Computations

### Resurgent analysis

In this section we briefly review some basic ingredients of resurgent analysis. A detailed exposition may be found for example in [ABS19, MS16]. Given a Gevrey-1 series

55 $$\begin{aligned} \varphi (\tau )=\sum _{n \ge 0} a_n \tau ^{n},\quad a_n = O(C^n n!), \end{aligned}$$

its Borel transform is defined by

56 $$\begin{aligned} {\widehat{\varphi }}(\zeta )= \sum _{k \ge 0} {a_k \over k!} \zeta ^k. \end{aligned}$$

It is a holomorphic function in a neighborhood of the origin. In favorable cases, this function can be extended to the complex $\zeta$-plane (also called Borel plane), but it will have singularities. Assuming that the analytically continued function does not grow too fast at infinity, the Borel resummation of $\varphi (\tau )$ is defined as the Laplace transform

57 $$\begin{aligned} s(\varphi )(\tau ) = \int _0^\infty \mathrm{e}^{-\zeta } {\widehat{\varphi }}(\zeta \tau ) \mathrm{d}\zeta . \end{aligned}$$

This has discontinuities at Stokes rays in the $\tau$ plane, whenever $\mathrm{arg}(\tau )= \mathrm{arg}(\zeta _s)$, where $\zeta _s$ is a singularity of ${\widehat{\varphi }}(\zeta )$. We define the lateral Borel resummations for $\tau$ near a Stokes ray by

58 $$\begin{aligned} s_\pm (\varphi )(\tau )= \int _0^{\mathrm{e}^{\pm \mathrm{i}\epsilon } \infty } \mathrm{e}^{-\zeta } {\widehat{\varphi }}(\zeta \tau ) \mathrm{d}\zeta . \end{aligned}$$

In the context of the theory of resurgence, we are usually given a collection of transseries $\Phi _\omega (\tau )$, where $\omega$ belongs to an indexing set. These transseries have the form

59 $$\begin{aligned} \Phi _\omega (\tau )=\mathrm{e}^{-V_\omega /\tau } \varphi _\omega (\tau ), \qquad \varphi _\omega (\tau ) \in \mathbb {C}[[\tau ]], \end{aligned}$$

where $V_\omega$ is the “action" associated to the sector $\omega$. The Borel resummation of the trans-series $\Phi _\omega (\tau )$ is defined by

60 $$\begin{aligned} s(\Phi _\omega )(\tau ) =\mathrm{e}^{-V_\omega /\tau } s(\varphi _\omega )(\tau ) \end{aligned}$$

(with suitable care for the constant term of $\varphi _\omega (\tau )$). To measure the discontinuity of Borel resummations across a Stokes ray, one introduces the Stokes automorphism ${\mathfrak {S}}$ as

61 $$\begin{aligned} s_+= s_- {\mathfrak {S}}. \end{aligned}$$

In our case, the singularities of ${\widehat{\varphi }}(\tau )$ are logarithmic branch points (i.e. we are dealing with so-called simple resurgent functions). In that case, the Stokes automorphism can be expressed as a (possibly infinite) linear combination of transseries,

62 $$\begin{aligned} {\mathfrak {S}} (\Phi _\omega )= \Phi _{\omega }+ \sum _{\omega '} \mathsf {S}_{\omega \omega '} \Phi _{\omega '}. \end{aligned}$$

The coefficients $\mathsf {S}_{\omega \omega '}$ are the Stokes constants (note that with this convention, their signs are opposite to e.g. the ones in [ABS19].) The singularities of ${\widehat{\varphi }}_\omega (\tau )$ occur at the points $V_{\omega '}-V_{\omega }$ for which $\mathsf {S}_{\omega \omega '} \not =0$.

In the case that we consider in this paper, the transseries are labeled by the critical point $\sigma$ and the multicovering $\mu \in {\mathbb {Z}}$, i.e. $\omega =(\sigma , \mu )$. If there is a singularity in the Borel plane of $\Phi _{\sigma ,\mu }$ located at

63 $$\begin{aligned} \iota _{\sigma ,\sigma '}^{(\mu ,\lambda )} = \text {CS}(\sigma ;\mu ) - \text {CS}(\sigma ';\lambda ), \end{aligned}$$

representing another transseries $\Phi _{\sigma ',\lambda }$, then the Borel resummation $s(\Phi _{\sigma ,\mu })(\tau )$ is discontinuous across the Stokes ray $\rho _{\theta }$ with $\theta = \arg \iota _{\sigma ,\sigma '}^{(\mu ,\lambda )}$, and the associated Stokes automorphism reads

64 $$\begin{aligned} s_{+}(\Phi _{\sigma ,\mu })(\tau ) = s_{-}(\Phi _{\sigma ,\mu })(\tau ) + \mathsf {S}_{\sigma ,\sigma '}^{(\mu ,\lambda )} s_{-}(\Phi _{\sigma ',\lambda })(\tau ), \end{aligned}$$

where $\mathsf {S}_{\sigma ,\sigma '}^{(\mu ,\lambda )}$ is the Stokes constant. Equation (2) implies that $\mathsf {S}_{\sigma ,\sigma '}^{(\mu ,\lambda )} = \mathsf {S}_{\sigma ,\sigma '}^{(\lambda -\mu )}$ depends on $\sigma ,\sigma '$ and the difference $\lambda -\mu$, an arbitrary integer number. It follows the equation of Stokes automorphism (64) can be written as

65 $$\begin{aligned} s_{+}(\Phi )(\tau ) = \mathfrak {S}_{\theta }({\tilde{q}}) s_{-}(\Phi )(\tau ) \end{aligned}$$

with $\Phi (\tau )=(\Phi _\sigma (\tau ))_\sigma$ the vector of asymptotic series, and the Stokes automorphism matrix

66 $$\begin{aligned} \mathfrak {S}_{\theta }({\tilde{q}}) = I + \mathsf {S}_{\sigma ,\sigma '}^{(k)}{\tilde{q}}^k E_{\sigma ,\sigma '} \end{aligned}$$

where $E_{\sigma ,\sigma '}$ is the elementary matrix with $(\sigma ,\sigma ')$-entry 1 and all other entries zero. Furthermore, all the Stokes constants are encoded in the two Stokes matrices

67 $$\begin{aligned} \mathsf {S}^+({\tilde{q}}) = \mathfrak {S}_{-\epsilon \rightarrow \pi -\epsilon }({\tilde{q}}),\quad \mathsf {S}^-({\tilde{q}}^{-1}) = \mathfrak {S}_{\pi -\epsilon \rightarrow 2\pi -\epsilon }({\tilde{q}}) \end{aligned}$$

(for $\epsilon >0$ and sufficiently small) where $\mathfrak {S}_{\theta ^-\rightarrow \theta ^+}$ is the global Stokes automorphism matrix defined for two non-Stokes rays whose arguments satisfy $0\le \theta ^+-\theta ^- \le \pi$ by

68 $$\begin{aligned} \mathfrak {S}_{\theta ^-\rightarrow \theta ^+}({\tilde{q}}) = \prod _{\theta ^-<\theta <\theta ^+}^{\longleftarrow } \mathfrak {S}_{\theta }({\tilde{q}}) \end{aligned}$$

where the ordered product is taken over the Stokes rays in the cone generated by $\rho _{\theta ^-}$ and $\rho _{\theta ^+}$. This factorization is well-known in the classical literature on the WKB method (see for example [Vor83] where it is called the “radar method”), and we will discuss it in more detail including its uniqueness in [GGMn20a]. Note the Stokes automorphisms are now represented by two finite-dimensional matrices $\mathsf {S}^+({\tilde{q}})$ and $\mathsf {S}^-({\tilde{q}}^{-1})$ in which the entries have been promoted to ${\tilde{q}}$ and ${\tilde{q}}^{-1}$-series respectively. This reorganization of the transseries is reminiscent of what was done in [CC01].

To numerically compute the integer coefficients of the above ${\tilde{q}}$-series, we need a high precision numerical computation of the Laplace integrals. And here lies the issue. In practice, only a few hundred coefficients of the series $\varphi (\tau )$ can be obtained. For instance, for the $4_1$ knot, the stationary phase of the state integral allows one to compute 300 coefficients of $\varphi _{\sigma _{1,2}}(\tau )$, and for the $5_2$ knot about 200 coefficients of $\varphi _{\sigma _{1,2,3}}(\tau )$ can be obtained. Alternatively, a numerical computation of the Kashaev invariant together with numerical extrapolation gives about 100 terms. Given such a truncated series, one can use Padé approximants to analytically continue the Borel transform to the complex plane, and then calculate the Borel resummation numerically. The Padé approximant can be also used to determine numerically the singularities in the Borel plane. Precision can be improved by using a conformal mapping, see [CMHR+07] for a summary of numerical techniques.

**Fig. 1:**
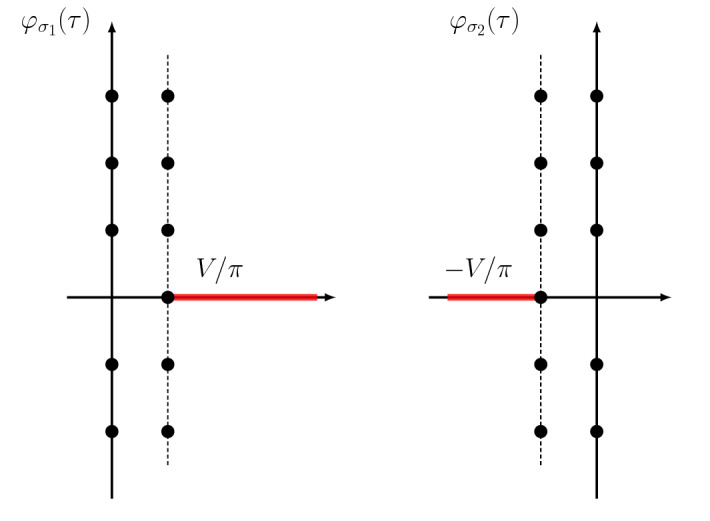
The singularities in the Borel plane for the series $\varphi _{\sigma _{1,2}} (\tau )$

### The $4_1$ knot

**Fig. 2:**
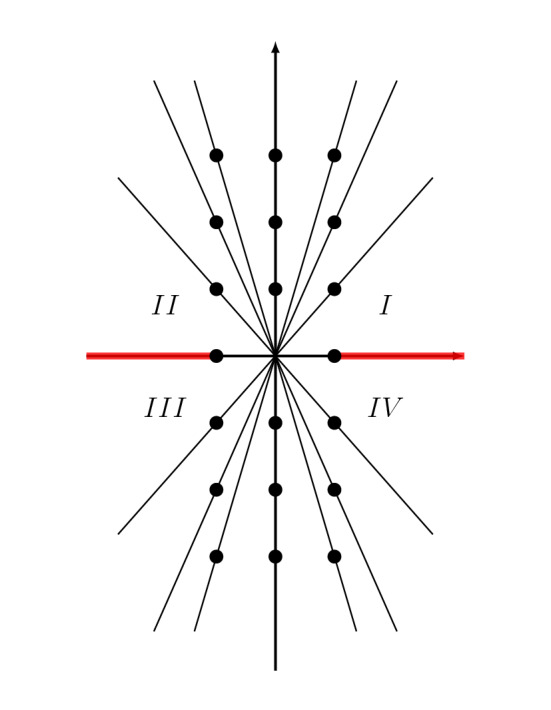
Stokes rays in the $\tau$-plane for $\Phi (\tau )$

The structure of singularities in the Borel plane for the formal power series $\varphi _{1,2}(\tau )$ of the $4_1$ knot is shown in Fig. 1. Points in each vertical line are $2\pi \mathrm{i}$ apart, and the two points in the real axis correspond to

69 $$\begin{aligned} \iota _{\pm } = \pm \left( {V(\sigma _1)\over 2 \pi } -{V(\sigma _2)\over 2 \pi } \right) = \pm {V \over \pi }, \end{aligned}$$

where *V* is defined in (9). Since each singularity in the Borel plane leads to a discontinuity in the Borel resummation, one has the structure of Stokes rays shown in Fig. 2 (where we took into account both series). Note that there is an infinite dense set of rays accumulating towards the imaginary axis.

We already hinted at the end of Sect. 4.2 that the asymptotic expansion (38) can be upgraded to an *exact* expression. To do this, one has to upgrade the optimal truncation of $\Phi (\tau )$ to its Borel resummation. Simultaneously we have to promote the matrix of constants appearing in  (38), to a matrix whose entries are power series in ${\tilde{q}}$ with integer coefficients:

70 $$\begin{aligned} \begin{pmatrix} \frac{1}{\sqrt{\tau }} g^0_0(\tau ) \\ \sqrt{\tau } g^1_0(\tau ) \end{pmatrix} = M_R ({\tilde{q}}) \, s_R(\Phi )(\tau ). \end{aligned}$$

The index *R* labels a sector in the $\tau$-plane, since due to presence of Stokes rays, both the matrix $M_R({\tilde{q}})$ and the Borel resummed vector $\Phi$ depend on the sector of the $\tau$-plane. In view of the structure of the Stokes rays, convenient sectors to perform the analysis are the angular wedges (i.e., pointed open cones in the complex plane) denoted by *I*, *II*, *III* and *IV* in Fig. 2.

It is a challenge to numerically compute the matrix $M_R({\tilde{q}})$ given only a few hundred terms of $\Phi (\tau )$, since the volume of $4_1$ (about $2.02\dots$) is so much smaller than the instanton corrections (appearing at $4\pi ^2 = 39.47\dots$). This can be done however, and with 300 terms of $\Phi (\tau )$ it is possible to compute the first twelve terms in the series appearing in $M_R({\tilde{q}})$. One finds for example, in region *I*,

71 $$\begin{aligned} M_I(q) = \begin{pmatrix} 1 - q - 2 q^2 - 2 q^3 - 2 q^4 &{} -1 + 2 q + 3 q^2 + 2 q^3 + q^4 \\ 1 - 7 q - 14 q^2 - 8 q^3 - 2 q^4 &{} 1 + 10 q + 15 q^2 - 2 q^3 - 19 q^4 \end{pmatrix} +O(q^5).\nonumber \\ \end{aligned}$$

Our Conjecture 1 suggests that these *q*-series can be expressed in terms of solutions to the linear *q*-difference equation (11). Indeed, one has, at this order,

72 $$\begin{aligned} M_I(q)= \begin{pmatrix} G_0^0 (q) &{} -G_0^0(q) - G_{-1}^0(q) \\ G_0^1 (q) &{} -G_0^1(q) - G_{-1}^1(q) \end{pmatrix} = W_{-1}(q)^T \, \begin{pmatrix} 0 &{} -1 \\ 1 &{} -1 \end{pmatrix} \,. \end{aligned}$$

We conjecture that this is in fact the exact expression for this matrix.

An important consequence of the relation (70) is that, by inverting it, one can express the Borel resummations of $\Phi _{\sigma _{j}}$ for $j=1,2$ in a given sector, in terms of the descendants of the state integral introduced in (4.3), which are holomorphic functions of $\tau$ on $\mathbb {C}'$. Indeed, let us consider the “reduced" descendant

73 $$\begin{aligned} {\widetilde{{\mathcal {I}}}}_{1,2,\lambda ,\mu }(\mathsf {b}) = \frac{\tau ^{1/2}}{2}G^1_\lambda (q)G^0_\mu ({\tilde{q}}) - \frac{\tau ^{-1/2}}{2}G^0_\lambda (q)G^1_\mu ({\tilde{q}}). \end{aligned}$$

This differs from the descendant (53) in a manifestly holomorphic factor, so it is holomorphic on $\mathbb {C}'$. Then, one finds, in region *I*,

74 $$\begin{aligned} s_I(\Phi _{\sigma _1})(\tau )= {\widetilde{{\mathcal {I}}}}_{1,2,0,0}(\tau ) + {\widetilde{{\mathcal {I}}}}_{1,2,0,-1,}(\tau ), \qquad s_I(\Phi _{\sigma _2})(\tau )= {\widetilde{{\mathcal {I}}}}_{1,2,0,0}(\tau ). \end{aligned}$$

This procedure can be done in the other sectors appearing in Fig. 2: one calculates $M_R(q)$, express it in terms of fundamental solutions, and represent the Borel resummation $s_R(\Phi )$ in terms of holomorphic functions on $\mathbb {C}'$. By comparing the different expressions for the Borel resummations in different sectors, one deduces the Stokes automorphisms relating them, and from a composition of the Stokes automorphisms one deduces the promised $\mathsf {S}$ matrices.

The results for $M_R(q)$ are the following:

75 $$\begin{aligned} \begin{aligned} M_{II}(q)&=\begin{pmatrix} G_0^0(q) + G_{-1}^0(q)&{} -G_0^0(q) \\ -G_0^1(q) - G_{-1}^1(q) &{} G_0^1 (q) \end{pmatrix} = \begin{pmatrix} 1 &{} 0 \\ 0 &{} -1 \end{pmatrix} \, W_{-1}(q)^T \, \begin{pmatrix} 1 &{} 0 \\ 1 &{}-1 \end{pmatrix} , \\ M_{III}(q)&=\begin{pmatrix} G_0^0(q^{-1}) + G_{-1}^0(q^{-1})&{} G_0^0(q^{-1}) \\ G_0^1(q^{-1}) +G_{-1}^1(q^{-1}) &{} G_0^1 (q^{-1}) \end{pmatrix} = W_{-1}(q^{-1})^T \, \begin{pmatrix} 1 &{} 0 \\ 1 &{} 1 \end{pmatrix},\\ M_{IV}(q)&=\begin{pmatrix} G_0^0(q^{-1}) &{} G_0^0(q^{-1}) + G_{-1}^0(q^{-1}) \\ -G_0^1 (q^{-1}) &{} -G_0^1(q^{-1}) -G_{-1}^1(q^{-1}) \end{pmatrix} =\begin{pmatrix} 1 &{} 0 \\ 0 &{} -1 \end{pmatrix} \, W_{-1}(q^{-1})^T \, \begin{pmatrix} 0 &{} 1 \\ 1 &{} 1 \end{pmatrix} . \end{aligned} \end{aligned}$$

From these values one deduces the Stokes automorphisms

76 $$\begin{aligned} s_{II}(\Phi )&= {\mathfrak {S}}_{I \rightarrow II}({\tilde{q}})s_{I}(\Phi ),&s_{IV}(\Phi )&= {\mathfrak {S}}_{III \rightarrow IV}({\tilde{q}}^{-1})s_{III}(\Phi )\nonumber \\ s_{I}(\Phi )&= \mathfrak {S}_{IV\mapsto I} s_{IV}(\Phi ),&s_{III}(\Phi )&= \mathfrak {S}_{II\mapsto III} s_{III}(\Phi ), \end{aligned}$$

where

77a $$\begin{aligned} {\mathfrak {S}}_{I \rightarrow II}(q)&= M_{II}(q)^{-1} M_I(q)&{\mathfrak {S}}_{III \rightarrow IV}(q)&= M_{IV}(q^{-1})^{-1} M_{III}(q^{-1}) \end{aligned}$$

77b $$\begin{aligned} {\mathfrak {S}}_{IV\rightarrow I}&= M_I(q)^{-1}M_{IV}(q)&{\mathfrak {S}}_{II\rightarrow III}&= M_{III}(q)^{-1}M_{II}(q) \,. \end{aligned}$$

and

78 $$\begin{aligned} \mathsf {S}^+(q)= {\mathfrak {S}}_{I \rightarrow II}(q) {\mathfrak {S}}_{IV\rightarrow I}, \qquad \mathsf {S}^-(q)= {\mathfrak {S}}_{III \rightarrow IV}(q) {\mathfrak {S}}_{II\rightarrow III}\,. \end{aligned}$$

Substituting the conjectured values for $M_R$ and using symmetry and the orthogonality relations  (15) and (16) one obtains (18a), (18b). In the $q\mapsto 0$ limit the Stokes matrices read

79 $$\begin{aligned} \mathsf {S}^+(0) = \begin{pmatrix} 1&{}3\\ 0&{}1 \end{pmatrix}\,,\quad \mathsf {S}^-(0) = \begin{pmatrix} 1&{}0\\ {}-3&{}1 \end{pmatrix}. \end{aligned}$$

The off–diagonal entries $\pm 3$ are Stokes constants associated to the singularities $\iota _{\pm }$ on the positive and negative real axis respectively. They agree with the matrix of integers obtained in [GH18, GZc]. Note that the Stokes matrices $\mathsf {S}^{\pm }(q)$ can also be factorized according to (67) in order to extract all the other Stokes constants. This will be studied in [GGMn20a]. Let us finally note that

80 $$\begin{aligned} \mathsf {S}^+_{\sigma _1 \sigma _1}(q) = G_0^0(q) G_0^1 (q) =1 -8 q -9 q^2 +18 q^3 + 46 q^4+ O(q^5) = \mathrm{Ind}^\mathrm{rot}_{4_1}(q),\qquad \end{aligned}$$

in agreement with Conjecture 2 (the fact that $$ G_0^0(q) G_0^1 (q)$$ equals the rotated index was pointed out in [GZb]).

### The $5_2$ knot

**Fig. 3:**
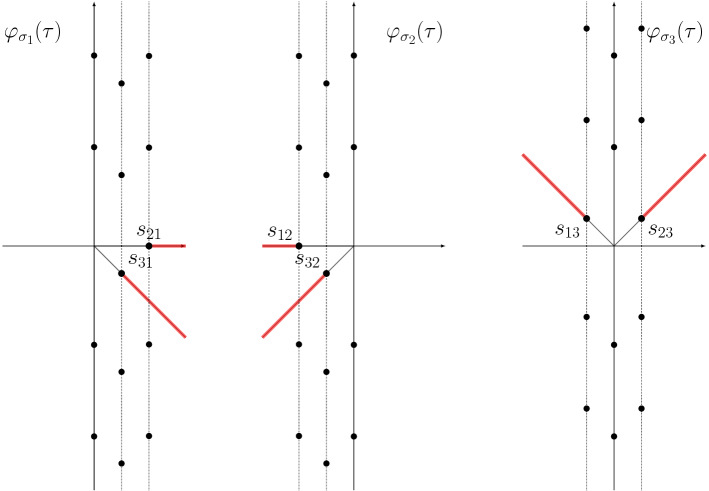
The singularities in the Borel plane for the series $\varphi _{\sigma _{j}} (\tau )$ of the $5_2$ knot for $j=1,2,3$

**Fig. 4:**
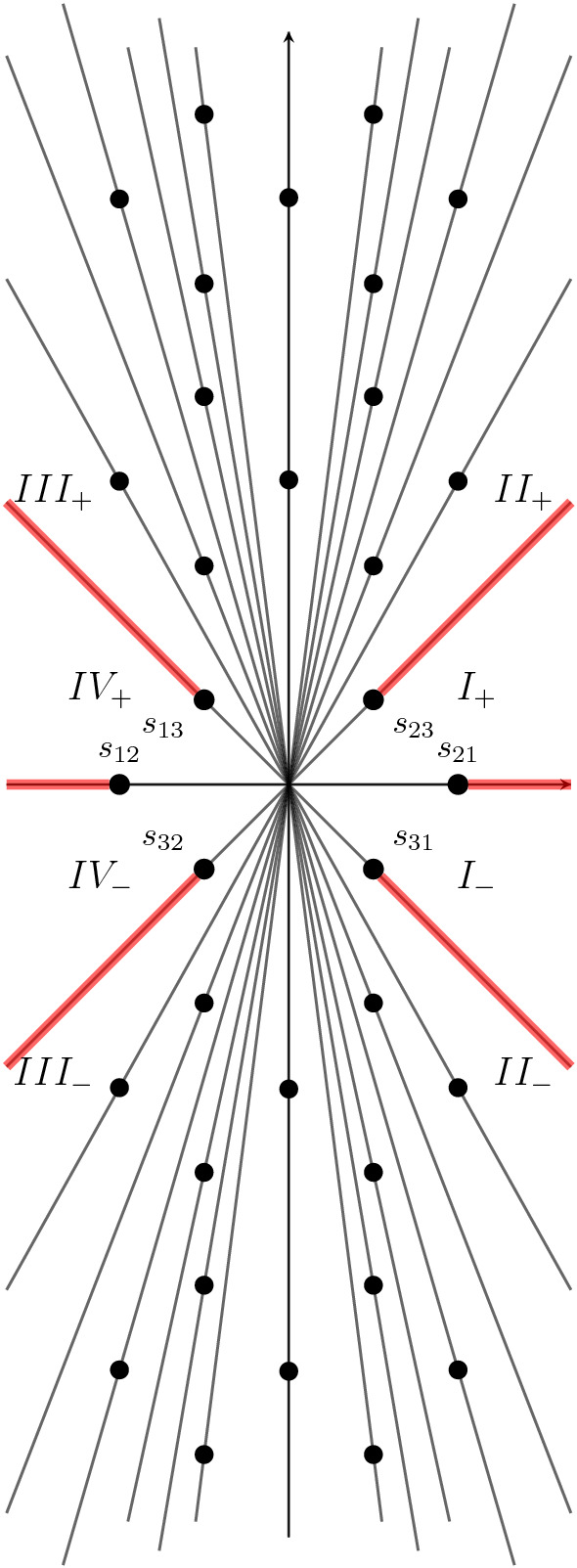
Stokes rays in the $\tau$-plane for $\Phi _{\sigma _{j}}(\tau )$ of the $5_2$ knot for $j=1,2,3$. Note that the points $s_{23},s_{31}$ happen to have the same real part, and so do $s_{13},s_{32}$. The dots are not shown in scale for aesthetic purpose

The structure of singularities in the Borel plane for the formal power series $\varphi _{\sigma _j}(\tau )$ ($j=1,2,3$) of the $5_2$ knot is shown in Fig. 3. Points in each vertical line are $2\pi \mathrm{i}$ are apart, while the six points $\iota _{ij}$ surrounding the origin are given by

81 $$\begin{aligned} \iota _{ij} = \frac{V(\sigma _i)}{2\pi } - \frac{V(\sigma _j)}{2\pi },\qquad 1 \le i\ne j \le 3 \,, \end{aligned}$$

where $V(\sigma _i)$ are given in (21). The structure of Stokes rays is shown in Fig. 4. There is also an infinite dense set of rays accumulating towards the imaginary axis.

The *q*-series $H^{\pm }_{k,0}$ for $(k=0,1,2)$, which are analogues of $G^0_0,G^1_0$ of the $4_1$ knot, have similarly interesting radial asymptotics. We use small letters for the corresponding functions, i.e.

82 $$\begin{aligned} h_k(\tau ) = {\left\{ \begin{array}{ll} H^+_{k,0}(\mathrm{e}^{2\pi \mathrm{i}\tau }),\quad &{} \mathfrak {I}(\tau ) > 0\\ H^-_{k,0}(\mathrm{e}^{-2\pi \mathrm{i}\tau }),\quad &{} \mathfrak {I}(\tau ) < 0 \end{array}\right. },\quad k=0,1,2 \,. \end{aligned}$$

Then the exact expression of the radial asymptotics reads

83 $$\begin{aligned} \mathrm{e}^{3\pi \mathrm{i}/4} \left( \begin{array}{c} \tau ^{-1} h_{0}(\tau )\\ h_{1}(\tau )\\ \tau h_{2}(\tau ) \end{array}\right) = M_R({\tilde{q}}) s_R(\Phi )(\tau ), \end{aligned}$$

where the index *R* labels a sector in the $\tau$-plane. The entries of the matrix $M_R({\tilde{q}})$ are power series in ${\tilde{q}}$ in the upper half plane, and power series in $1/{\tilde{q}}$ in the lower half-plane.

With more than 200 terms of $\Phi (\tau )$, we are able to compute first few terms in $M_R({\tilde{q}})$. For instance, in region $I_+$ we are able to compute first six terms in each entry of $M_{I_+}({\tilde{q}})$. We display some of the results here

84 $$\begin{aligned} M_{I_+}(q) = \begin{pmatrix} -1 - q^2 &{} 2 + 3 q^2 &{} 1 + q + 3 q^2 \\ -1 + 3q + 3q^2 &{} 1 - 6q - 3 q^2 &{}-q \\ -\frac{5}{6} + 5 q -\frac{53}{6}q^2&{} -\frac{4}{3} - 4q + \frac{77}{2}q^2 &{}-\frac{1}{6} + \frac{29}{6}q +\frac{55}{2}q^2 \end{pmatrix} +O(q^3). \end{aligned}$$

Following Conjecture 1, we can express it in terms of solutions to the linear *q*-difference equation (23)

85 $$\begin{aligned} M_{I_+}(q) = W_{-1}(q)^T \begin{pmatrix} 0&{}0&{}1\\ -1&{}3&{}0\\ 0&{}-1&{}0 \end{pmatrix}, \end{aligned}$$

where the Wronskian is defined in (25). Note that this expression is exact. By inverting the matrix $M_{I_+}(q)$, we can express the Borel resummation of $\Phi _{\sigma _{j}}$ for $j=1,2,3$ in the sector $I_+$ in terms of the descendants of the state integal introduced in Eq. (39). Let us again introduce the “reduced” descendant

86 $$\begin{aligned} \widetilde{\mathcal {I}}_{\lambda ,\mu }(\tau ) = \tau H_{2,\lambda }^{+}(q)H_{0,\mu }^{-}({\tilde{q}}) -2 H_{1,\lambda }^{+}(q)H_{1,\mu }^{-}({\tilde{q}}) +\tau ^{-1} H_{0,\lambda }^{+}(q)H_{2,\mu }^{-}({\tilde{q}}), \end{aligned}$$

which differs from the descendant (111) in a manifestly holomorphic factor. We find in region $I_+$

87 $$\begin{aligned} s_{I_+}(\Phi )(\tau ) = \frac{1}{2}\mathrm{e}^{3\pi \mathrm{i}/4} \begin{pmatrix} -1 &{} -1 &{} 0 \\ 0 &{} -1 &{} 0\\ 0 &{} 0 &{} 1 \end{pmatrix} \left( \begin{array}{c} \widetilde{\mathcal {I}}_{0,-1}\\ \widetilde{\mathcal {I}}_{0,0}\\ \widetilde{\mathcal {I}}_{0,1}\\ \end{array}\right) (\tau ). \end{aligned}$$

In other regions, the results for $M_R(q)$ are as follows:

- In the upper half-plane
  88 $$\begin{aligned} M_{II_+}(q) =&W_{-1}(q)^T \begin{pmatrix} 0&{}-3&{}1\\ -1&{}3&{}0\\ 0&{}-1&{}0 \end{pmatrix}, \end{aligned}$$
  89 $$\begin{aligned} M_{III_+}(q) =&\begin{pmatrix} 1&{}0&{}0\\ 0&{}-1&{}0\\ 0&{}0&{}1 \end{pmatrix} W_{-1}(q)^T \begin{pmatrix} -3&{}0&{}1\\ 3&{}-1&{}0\\ -1&{}0&{}0 \end{pmatrix}, \end{aligned}$$
  90 $$\begin{aligned} M_{IV_+}(q) =&\begin{pmatrix} 1&{}0&{}0\\ 0&{}-1&{}0\\ 0&{}0&{}1 \end{pmatrix} W_{-1}(q)^T \begin{pmatrix} 0&{}0&{}1\\ 3&{}-1&{}0\\ -1&{}0&{}0 \end{pmatrix},\qquad |q|<1. \end{aligned}$$
- In the lower half-plane:
  91 $$\begin{aligned} M_{I_-}(q) =&\begin{pmatrix} 1&{}0&{}0\\ 0&{}-1&{}0\\ 0&{}0&{}1 \end{pmatrix}W_{-1}(q)^T \begin{pmatrix} 0&{}0&{}1\\ -1&{}-1&{}0\\ 0&{}-1&{}0 \end{pmatrix}, \end{aligned}$$
  92 $$\begin{aligned} M_{II_-}(q) =&\begin{pmatrix} 1&{}0&{}0\\ 0&{}-1&{}0\\ 0&{}0&{}1 \end{pmatrix}W_{-1}(q)^T \begin{pmatrix} 0&{}0&{}1\\ -1&{}-1&{}-3\\ 0&{}-1&{}0 \end{pmatrix},\end{aligned}$$
  93 $$\begin{aligned} M_{III_-}(q) =&W_{-1}(q)^T \begin{pmatrix} 0&{}0&{}1\\ -1&{}-1&{}-3\\ -1&{}0&{}0 \end{pmatrix}, \end{aligned}$$
  94 $$\begin{aligned} M_{IV_-}(q) =&W_{-1}(q)^T \begin{pmatrix} 0&{}0&{}1\\ -1&{}-1&{}0\\ -1&{}0&{}0 \end{pmatrix},\qquad |q|>1 . \end{aligned}$$

From these values one can deduce the Stokes automorphism, in the anticlockwise direction

95 $$\begin{aligned} s_{III_+}(\Phi ) = \mathfrak {S}_{II_+\rightarrow III_+}({\tilde{q}}) s_{II_+}(\Phi ),\quad s_{II_-}(\Phi ) = \mathfrak {S}_{III_-\rightarrow II_-}({\tilde{q}}^{-1}) s_{III_-}(\Phi ),\nonumber \\ s_{II_+}(\Phi ) = \mathfrak {S}_{I_+\rightarrow II_+} s_{I_+}(\Phi ),\quad s_{I_-}(\Phi ) = \mathfrak {S}_{II_-\rightarrow I_-} s_{II_-}(\Phi ),\nonumber \\ s_{IV_+}(\Phi ) = \mathfrak {S}_{III_+\rightarrow IV_+} s_{III_+}(\Phi ),\quad s_{III_-}(\Phi ) = \mathfrak {S}_{IV_-\rightarrow III_-} s_{IV_-}(\Phi ),\nonumber \\ s_{I_+}(\Phi ) = \mathfrak {S}_{I_-\rightarrow I_+} s_{I_-}(\Phi ),\quad s_{IV_-}(\Phi ) = \mathfrak {S}_{IV_+\rightarrow IV_-} s_{IV_+}(\Phi ), \end{aligned}$$

where

96 $$\begin{aligned} \mathfrak {S}_{II_+\rightarrow III_+}(q) =&\frac{1}{2} \begin{pmatrix} 0&{}-1&{}0\\ 0&{}-1&{}-1\\ 1&{}-3&{}0 \end{pmatrix}\cdot W_{-1}(q^{-1})\cdot \begin{pmatrix} 0&{}0&{}1\\ 0&{}-2&{}0\\ 1&{}0&{}0 \end{pmatrix}\cdot W_{-1}(q)\cdot \begin{pmatrix} 0&{}-3&{}1\\ -1&{}3&{}0\\ 0&{}-1&{}0 \end{pmatrix}, \end{aligned}$$

97 $$\begin{aligned} \mathfrak {S}^{-1}_{III_-\rightarrow II_-}(q) =&\frac{1}{2} \begin{pmatrix} 0&{}-1&{}0\\ -3&{}3&{}-1\\ 1&{}0&{}0 \end{pmatrix} \cdot W_{-1}(q) \cdot \begin{pmatrix} 0&{}0&{}1\\ 0&{}-2&{}0\\ 1&{}0&{}0 \end{pmatrix}\cdot W_{-1}(q^{-1})^T \cdot \begin{pmatrix} 0&{}0&{}1\\ -1&{}-1&{}-3\\ 0&{}-1&{}0 \end{pmatrix}, \end{aligned}$$

and $\mathfrak {S}_{I_-\rightarrow I_+}$,$\mathfrak {S}_{I_+\rightarrow II_+}$,$\mathfrak {S}_{III_+\rightarrow IV_+}$,$\mathfrak {S}_{IV_+\rightarrow IV_-}$,$\mathfrak {S}_{IV_-\rightarrow III_-}$,$\mathfrak {S}_{II_-\rightarrow I_-}$ are given in (100a),(100b). The matrices $\mathsf {S}^{\pm }(q)$ are simply

98 $$\begin{aligned} \mathsf {S}^+(q) =&\mathfrak {S}_{III_+\rightarrow IV_+} \mathfrak {S}_{II_+\rightarrow III_+}(q)\mathfrak {S}_{I_+\rightarrow II_+}\mathfrak {S}_{I_-\rightarrow I_+}, \end{aligned}$$

99 $$\begin{aligned} \mathsf {S}^-(q) =&\mathfrak {S}_{II_-\rightarrow I_-}\mathfrak {S}_{III_-\rightarrow II_-}(q)\mathfrak {S}_{IV_-\rightarrow III_-} \mathfrak {S}_{IV_+\rightarrow IV_-}, \end{aligned}$$

and we obtain (29a),(29b).

In the $q\mapsto 0$ limit, the Stokes matrices factorize

100a $$\begin{aligned} \mathsf {S}^+(0)\,=\,&\mathfrak {S}_{\sigma _3,\sigma _1}\mathfrak {S}_{\sigma _3,\sigma _2}\mathfrak {S}_{\sigma _1,\sigma _2} \,=\, \begin{pmatrix} 1&{}0&{}0\\ 0&{}1&{}0\\ {}-3&{}0&{}1 \end{pmatrix} \begin{pmatrix} 1&{}0&{}0\\ 0&{}1&{}0\\ 0&{}3&{}1 \end{pmatrix} \begin{pmatrix} 1&{}4&{}0\\ 0&{}1&{}0\\ 0&{}0&{}1 \end{pmatrix}, \end{aligned}$$

100b $$\begin{aligned} \mathsf {S}^-(0) =&\mathfrak {S}_{\sigma _1,\sigma _3}\mathfrak {S}_{\sigma _2,\sigma _3}\mathfrak {S}_{\sigma _2,\sigma _1} = \begin{pmatrix} 1&{}0&{}3\\ 0&{}1&{}0\\ 0&{}0&{}1 \end{pmatrix} \begin{pmatrix} 1&{}0&{}0\\ 0&{}1&{}-3\\ 0&{}0&{}1 \end{pmatrix} \begin{pmatrix} 1&{}0&{}0\\ {}-4&{}1&{}0\\ 0&{}0&{}1 \end{pmatrix}. \end{aligned}$$

The non-vanishing off-diagonal entry of $\mathfrak {S}_{\sigma _i,\sigma _j}$ is the Stokes constant associated to the Borel singularity $\iota _{i,j}$. Assembling these Stokes constants in a matrix we obtain

101 $$\begin{aligned} \begin{pmatrix} 0&{}4&{}3\\ -4&{}0&{}-3\\ -3&{}3&{}0 \end{pmatrix} \end{aligned}$$

which is what was found numerically in [GZc, Sec.3.3]. Note that the Stokes matrices $\mathsf {S}^{\pm }(q)$ can also be factorized according to (67) in order to extract all the other Stokes constants. This will be studied in detail in [GGMn20a].

We note that

102 $$\begin{aligned} \mathsf {S}^+_{\sigma _1 \sigma _1}(q) = 2H_{1,0}^+(q) H_{1,0}^- (q) =1 -12 q +3 q^2 +74 q^3 + 90 q^4+ O(q^5) = \mathrm{Ind}^\mathrm{rot}_{5_2}(q),\nonumber \\ \end{aligned}$$

in agreement with Conjecture 2.

## Open questions

In this paper we have formulated conjectures on the full resurgent structure of quantum knot invariants of hyperbolic knots, and we have presented detailed evidence for the first non-trivial cases, namely the knots $4_1$ and $5_2$. Although we used complex Chern–Simons theory as a way to motivate our results, and state integrals and asymptotic series as a way to present them, it is clear that a key ingredient that controls the description of the asymptotic series in Borel plane is a pair of linear *q*-difference equations with explicit fundamental solutions. It is natural to ask whether these linear *q*-difference equations are related to those that annihilate the 3D-index, or the colored Jones polynomial of a knot [GL05]. The latter is the famous ${\widehat{A}}$-polynomial of a knot, whose specialization at $q=1$ is conjectured to essentially coincide with the *A*-polynomial of a knot [Gar04]. It is an interesting question to relate the newly found linear *q*-difference equations with the ${\widehat{A}}$-polynomial of a knot.

One could also consider deformations by an arbitrary holonomy around the knot, which will be explored in [GGMn20a]. In this case, the resulting perturbative series depend on a parameter *x* (see e.g. [DGLZ09]) that plays the role of a Jacobi variable and one could calculate the Stokes constants in this extended setting. This might make clearer the relation to the *A*-polynomial and its quantization.

Another interesting question is whether the Stokes constants we compute, which are closely related to BPS counting, can be obtained with techniques similar to those of [GMN13], i.e. by doing WKB analysis on the algebraic curve defined by the *A*-polynomial, or some variant thereof.

Finally, we would like to point out that towers of singularities similar to those studied here appear in the Borel plane of topological string partition functions, see e.g. [PS10, CSMnS17]. Understanding the Stokes constants of these singularities in topological string theory would probably lead to fascinating mathematics and to connections with BPS state counting in string theory.

## Appendix A. *q*-Series Associated with the $5_2$ Knot

### A.1 The state integral of the $5_2$ knot

We now consider the case of $(A,B)=(2,3)$, i.e., the state integral ${\mathcal {I}}_{2,3,\lambda ,\mu }(\mathsf {b})$. The data we need to present our result are

103 $$\begin{aligned}&F_{2,3,\lambda }(q,x) = \sum _{n=0}^\infty \frac{q^{n(n+1)+n\lambda }}{(q;q)_n^3}x^n, \end{aligned}$$

104 $$\begin{aligned}&\widetilde{F}_{2,3,\mu }({\tilde{q}},{\tilde{x}}) = \sum _{n=0}^\infty (-1)^n \frac{{\tilde{q}}^{\frac{1}{2}n(n+1)+n\mu }}{({\tilde{q}};{\tilde{q}})_n^3}{\tilde{x}}^n, \end{aligned}$$

as well as the operator

105 $$\begin{aligned} P_{2,3,\lambda ,\mu } =&-\frac{1}{2\pi \mathrm{i}}+(1+2\delta -3\delta _1+\lambda ) (\frac{1}{2}+{\tilde{\delta }} -3{\tilde{\delta }}_1 + \mu ) \end{aligned}$$

106 $$\begin{aligned}&-\frac{\mathsf {b}^2}{2}((1+2\delta -3\delta _1+\lambda )^2-3\delta _2) \end{aligned}$$

107 $$\begin{aligned}&-\frac{\mathsf {b}^{-2}}{2}((\frac{1}{2}+{\tilde{\delta }} -3{\tilde{\delta }}_1 + \mu )^2-\frac{1}{4}-3{\tilde{\delta }}_2 + 6\, E_2^{(0)}({\tilde{q}})). \end{aligned}$$

Using the observation that

108 $$\begin{aligned} -\frac{1}{2\pi \mathrm{i}} = \frac{1}{12}\left( \tau E_2(\tau ) - \tau ^{-1}E_2(-1/\tau )\right) \end{aligned}$$

as well as

109 $$\begin{aligned} E_2(\tau ) = 1-24\sum _{s=1}^\infty \frac{q^s}{(1-q^s)^2} = 1-24 E_2^{(0)}(q), \end{aligned}$$

the final result can be written as ($\tau = \mathsf {b}^2$ in the upper half-plane)

110 $$\begin{aligned} {\mathcal {I}}_{2,3,\lambda ,\mu }(\mathsf {b})&= (-1)^{\lambda -\mu } e^{\frac{\pi \mathrm{i}}{4}}q^{\frac{\lambda }{2}}{\tilde{q}}^{\frac{\mu }{2}} \left( \frac{q}{{\tilde{q}}}\right) ^{\frac{1}{8}} \end{aligned}$$

111 $$\begin{aligned}&\quad \times \left( -\frac{\tau }{2}H_{2,\lambda }^{+}(q)H_{0,\mu }^{-}({\tilde{q}}) +H_{1,\lambda }^{+}(q)H_{1,\mu }^{-}({\tilde{q}}) -\frac{\tau ^{-1}}{2}H_{0,\lambda }^{+}(q)H_{2,\mu }^{-}({\tilde{q}})\right) . \end{aligned}$$

Here

112 $$\begin{aligned} H_{j,\lambda }^{+}(q) = \sum _{n=0}^\infty p_{n,\lambda }^{(j)}(q)t_{n,\lambda }(q),\quad H_{j,\mu }^{-}({\tilde{q}}) = \sum _{n=0}^\infty P_{n,\mu }^{(j)}({\tilde{q}}) T_{n,\mu }({\tilde{q}}),\qquad (j=0,1,2) \,,\nonumber \\ \end{aligned}$$

where $t_{n,\lambda }(q),T_{n,\mu }({\tilde{q}})$ are coefficients of $F_{2,3,\lambda }(q,x), \widetilde{F}_{2,3,\mu }({\tilde{q}},{\tilde{x}})$ as series of $x,{\tilde{x}}$,

113 $$\begin{aligned}&t_{n,\lambda }(q) = \frac{q^{n(n+1)+n\lambda }}{(q;q)_n^3}, \end{aligned}$$

114 $$\begin{aligned}&T_{n,\mu }({\tilde{q}}) = (-1)^n\frac{{\tilde{q}}^{\frac{1}{2}n(n+1)+n\mu }}{({\tilde{q}};{\tilde{q}})_n^3}, \end{aligned}$$

while $p_{n,\lambda }^{(j)}(q), P_{n,\mu }^{(j)}({\tilde{q}})$ result from applying the operator $P_{2,3,\lambda ,\mu }$, i.e.

115 $$\begin{aligned}&p_{n,\lambda }^{(0)}(q) = 1, \end{aligned}$$

116 $$\begin{aligned}&p_{n,\lambda }^{(1)}(q) = 1+2n-3E_1^{(n)}(q) + \lambda , \end{aligned}$$

117 $$\begin{aligned}&p_{n,\lambda }^{(2)}(q) = \left( p_{n,\lambda }^{(1)}(q)\right) ^2 - \frac{1}{6} - 3 E_2^{(n)}(q) + 4 E_2^{(0)}(q), \end{aligned}$$

as well as

118 $$\begin{aligned}&P_{n,\mu }^{(0)}({\tilde{q}}) = 1, \end{aligned}$$

119 $$\begin{aligned}&P_{n,\mu }^{(1)}({\tilde{q}}) = \frac{1}{2}+n-3E_1^{(n)}({\tilde{q}}) + \mu , \end{aligned}$$

120 $$\begin{aligned}&P_{n,\mu }^{(2)}({\tilde{q}}) = \left( P_{n,\mu }^{(1)}({\tilde{q}})\right) ^2 - \frac{1}{12} - 3 E_2^{(n)}({\tilde{q}}) + 2 E_2^{(0)}({\tilde{q}}). \end{aligned}$$

The *q*-series $H_{j,\lambda }^{+}(q)$ and $H_{j,\mu }^{-}(q)$ for $j=0,1,2$ are exactly those that appear in Eq. (25). A few coefficients of the above series are given by

### A.2. The symmetry $q \leftrightarrow q^{-1}$

We now discuss the symmetry $q \mapsto q^{-1}$. It is easy to see that the above *q*-series $H_{j,\lambda }^{\pm }(q)$ are well-defined holomorphic functions when *q* is either inside or outside the unit disk. Extended this way, we claim that

121 $$\begin{aligned} H_{j,\lambda }^{+}(q) = (-1)^j H_{j,-\lambda }^{-}(1/q), \qquad (q \in \mathbb {C},\,\, |q| \ne 1) \,. \end{aligned}$$

This follows from the easy observation

122 $$\begin{aligned} t_{n,\lambda }(q) = T_{n,-\lambda }(1/q) \, \end{aligned}$$

and the less trivial symmetry

123 $$\begin{aligned} E_1(\tau ) = -E_1(-\tau ),\quad E_2(\tau ) = -E_2(-\tau ) \end{aligned}$$

(see for instance [BC13] for the first and [CMZ18] for the second), where $E_1,E_2$ are related to $E_1^{(0)},E_2^{(0)}$ by

124 $$\begin{aligned} E_1(\tau )&= 1 - 4\sum _{n\ge 1} \frac{q^n}{1-q^n} = 1- 4 E_1^{(0)}(q), \end{aligned}$$

125 $$\begin{aligned} E_2(\tau )&= 1 - 24\sum _{n\ge 1} \frac{q^n}{(1-q^n)^2} = 1-24E_2^{(0)}(q) \,. \end{aligned}$$

Equations (125) and (126) and induction on the exponent *n* of $E_{1}^{(n)}, E_{2}^{(n)}$ imply that

126 $$\begin{aligned} p_{n,\lambda }^{(k)}(q) = (-1)^k P_{n,-\lambda }^{(k)}(1/q) \end{aligned}$$

for all *n*, and this concludes the proof of Eq. (124).

The above conclusions hold not only for the state integral with $(A,B)=(2,3)$ or $(A,B)=(1,2)$, but for the case of arbitrary integers *A* and *B* with $B> A > 0$.
